# A novel thermophilic strain of *Bacillus subtilis* with antimicrobial activity and its potential application in solid-state fermentation of soybean meal

**DOI:** 10.1128/spectrum.02784-23

**Published:** 2024-02-20

**Authors:** Nanshan Qi, Xiaoshu Zhan, Joshua Milmine, Kai-Hsiang Chang, Julang Li

**Affiliations:** 1 Institute of Animal Health, Guangdong Academy of Agricultural Sciences, Guangzhou, Guangdong, China; 2 Department of Animal Biosciences, University of Guelph, Guelph, Canada; 3 Department of Life Science and Engineering, Foshan University, Foshan, Guangdong, China; The Hebrew University of Jerusalem, Jerusalem, Israel

**Keywords:** *Bacillus subtilis*, antimicrobial activity, thermophilic, soybean meal, solid-state fermentation

## Abstract

Soybean meal (SBM) is the most important source of plant protein in animal feeds, containing around 41%–48% crude protein. Nevertheless, 70%–80% of these proteins is allergenic antigens that can have adverse implications for the gastrointestinal well-being of animals, especially to young animals. Microbial fermentation is one of the most cost-effective strategies used to reduce allergenic antigens from plant sources. In this study, we report the isolation and characterization of a novel probiotic *Bacillus subtilis* “L5” strain from lake mud. L5 demonstrated remarkable temperature tolerance across a broad temperature spectrum, thriving at 25°C, 37°C, and 50°C. In addition, antimicrobial assay revealed that L5 exhibits strong antimicrobial activity against *Escherichia coli*, effectively reducing or eliminating the growth of Gram-negative bacteria in SBM when fermented with L5. When applied to SBM fermentation, L5 efficiently reduced SBM antinutritional factors such as glycinin, β-conglycinin, trypsin inhibitor, phytic acid, neutral detergent fiber, and acid detergent fiber, which in turn results in an increase in crude protein content and the free amino acid concentration. Our findings on the probiotic and fermentation capabilities of L5 suggest that this novel bacterium has dual functions that make it a strong candidate for improving the nutrient values of feed via its role in fermentation.

**IMPORTANCE:**

Soybean meal (SBM), containing 41%–48% crude protein, is the most important source of plant protein in animal feeds. Unfortunately, 70%–80% of the proteins in SBM is allergenic antigens including trypsin inhibition, β-conglycinin, and conglycinin, which negatively affect intestine health and function. Microbial solid-state fermentation methods have been applied to animal feeds for decades, to eliminate antinutritional factors. Here, a novel potential probiotic *Bacillus subtilis* “L5” strain with high enzymatic activity and antimicrobial activity will be a great help to improve the quality and reproducibility of SBM fermentation.

## INTRODUCTION

Soybean meal (SBM), containing 41%–48% crude protein, is the most important source of plant protein in animal feeds (1). Unfortunately, 70%–80% of SBM proteins are allergenic antigens. These antigens include trypsin inhibitors, β-conglycinin, and conglycinin; all of which negatively affect intestine health and function (2). Microbial solid-state fermentation methods have been applied to animal feeds for decades, aiming to eliminate antinutritional factors (3). Recently, a large variety of microorganisms including fungi (*Aspergillus* spp., *Rhizopus oryzae* spp., and *Saccharomyces* spp.) and bacteria (*Bacillus* spp., *Lactobacillus* spp., *Bifidobacterium* spp., and *Shewanella* spp.) have been used in feed fermentation for nutritional enhancement (1). Previous studies showed that 48 hours of SBM fermentation with *Aspergillus niger* significantly reduced trypsin inhibitors (4). In another study, 24 hours of SBM fermentation with *Bacillus subtilis* BS12 reduced glycinin and β-conglycinin by 84.77% and 87.07%, respectively (5). Another 48 hours of SBM fermentation with *Shewanella* sp. MR-7 also showed a decrease in trypsin inhibitor, glycinin, and β-conglycinin by 90.19%, 77.36%, and 84.52%, respectively (6). These studies have suggested that screening of microorganism strains with high protease, cellulase, amylase, and xylanase activities is the key to developing an efficient fermentation.

The presence of undesirable bacteria within the fermentation substrate (feedstuff) presents a formidable challenge, as these inadvertent microbials can deplete substantial quantities of nutrients in SBM and can potentially outcompete the functional microorganism intended for fermentation (7). The strategies to mitigate undesired bacterial contamination during fermentation involve using pre-sterilized SBM or employing anaerobic bacteria for the fermentation process (8). However, these approaches necessitate intricate procedures, incur high energy expenditures, and demand specialized equipment, thereby rendering the feasibility of such methods. Therefore, a microorganism that elicits antimicrobial activity and is capable of eliminating other undesirable bacteria within the fermentation system can prove to be an advantageous alternative.

Additionally, the temperature of the fermentation vessel serves as a pivotal factor for a favorable fermentation. Previous studies suggest the optimal fermentation temperature that eliminates or inhibits the growth of the majority of unfavorable bacteria is usually at around 50°C (9). Therefore, screening and identification of a thermophilic bacterial strain, endowed with robust enzymatic and antimicrobial activities, are vital to improving the quality and reproducibility of SBM fermentation.

In the present study, a novel potential probiotic *B. subtilis* L5 strain was isolated. The growth properties of L5 were examined at various temperature ranges, and *in vitro* studies were conducted to assess the enzymatic and antimicrobial activities of L5. The application and method development of L5 in solid-state fermentation of SBM were analyzed as well.

## RESULTS

### Isolation of a novel thermophilic strain of *B. subtilis* L5

Screening of a lake mud sample from Saskatoon allowed us to recover 24 bacterial isolates. MALDI-TOF MS revealed that five isolates are from a bacterial family that has probiotic potential (*Bacillus* spp. L1, *B. cereus* L2, *B. pumilus* L3, *S. putrefaciens* L4 and *B. subtilis* L5).

**Fig 1:**
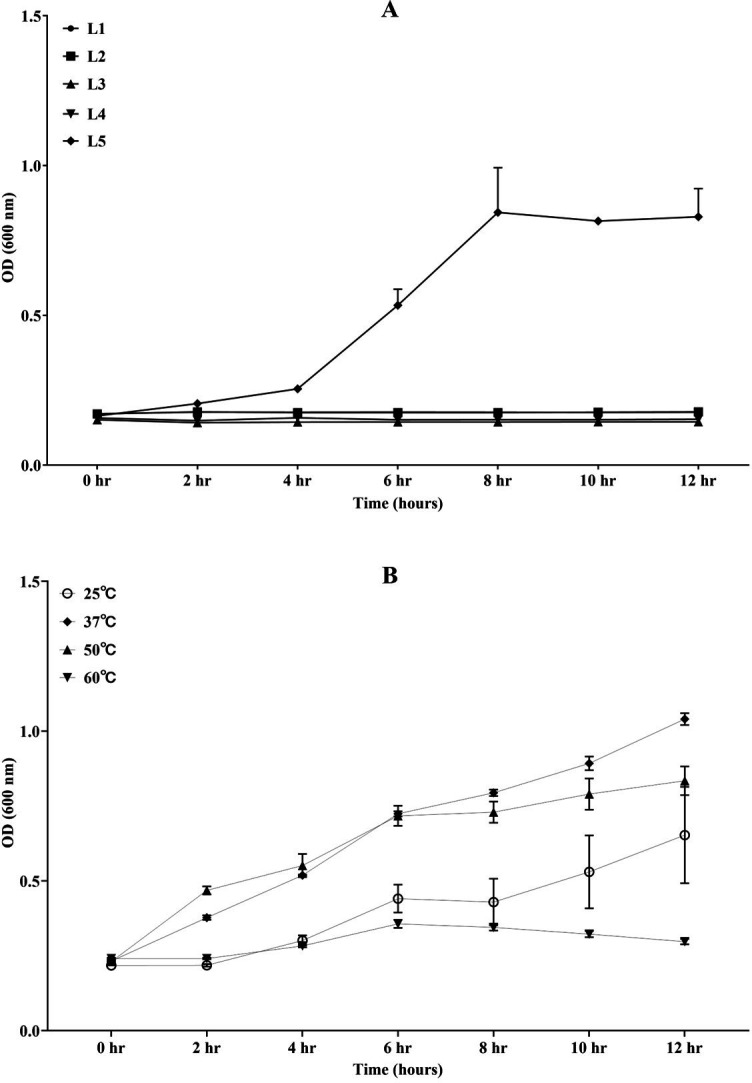
Growth characteristics of *Bacillus subtilis* L5 at different temperatures. (A) The growth curve of *Bacillus* spp. L1, *B. cereus* L2, *Bacillus pumilus* L3, *Shewanella putrefaciens* L4, and *B. subtilis* L5 incubated at 50°C for 12 hours. (B) The growth curve of L5 when it was incubated at 25°C, 37°C, 50°C, and 60°C for 12 hours. Error bars represent the SEM of the mean of three independent experiments.

To screen bacterial strains capable of high-temperature tolerance, the growth performance of L1–5 at 50°C was conducted. As shown in Fig. 1A, *B. subtilis* L5 was the only bacteria that grew well at 50°C, where it reached its exponential phase at four hours of culture and reached a plateau at ~8 hours while the other four stains could not grow at 50°C.

Upon culturing across a temperature range of 25°C to 60°C, L5 exhibited proficient growth at 25°C, 37°C, and 50°C, while growth was not obviously observed at 60°C. Notably, the growth dynamics of L5 varied at 37°C and 50°C within different time frames; L5 demonstrated a rapid initial growth at 50°C during the first 6 hours, whereas from 6 to 12 hours, a higher proliferation rate was observed at 37°C. Additionally, both at 37°C and 50°C, L5 displayed a significantly enhanced growth rate in comparison to its growth at 25°C and the non-permissive condition at 60°C (Fig. 1B).

### L5 is capable of protease, cellulase, amylase, and xylanase production

**Fig 2:**
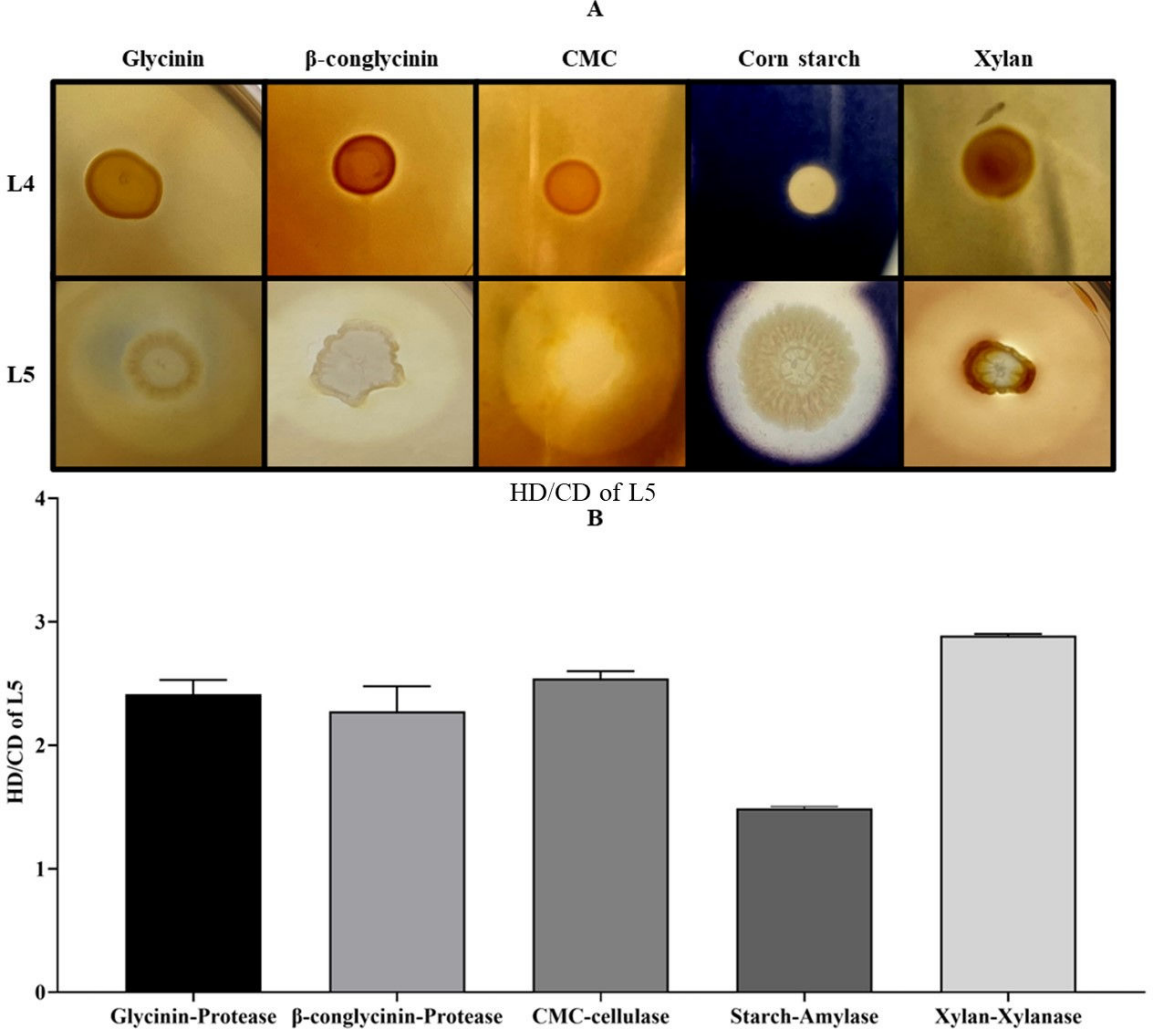
Detection of protease, cellulase, amylase, and xylanase activities for *Bacillus subtilis* L5 using glycinin/β-conglycinin-, CMC-, corn starch-, and xylan-supplemented agar plates. One microliter of *S. putrefaciens* L4 (Control) or *B. subtilis* L5 overnight culture broth was added on the agar plates containing different anti-nutritional factors and then was incubated at 37°C for 12 hours. (A) Clear zones on glycinin-, β-conglycinin-, CMC-, corn starch-, and xylan-supplemented plates; (B) HD/CD of L5. HD, diameter of clear zone; CD, diameter of colony. Error bars represent the SEM of the mean of three independent observations.

As shown in Fig. 2, the visible clear zone on the glycinin- and β-conglycinin-supplemented agar plates indicated that L5 has protease activity against these SBM allergens (Fig. 2A and B), in which the hydrolysis capacity (HC) [diameter of clear zone (HD)/diameter of colony (CD)] values were 2.41 ± 0.11 and 2.27 ± 0.20, respectively (Fig. 2E). The visible clear zone on the carboxymethyl cellulose (CMC) fiber-, corn starch-, and xylan-supplemented agar plates demonstrated that L5 has cellulase, amylase, and xylanase activities (Fig. 2A), in which the HC (HD/CD) values were 2.54 ± 0.06, 1.49 ± 0.02, and 2.89 ± 0.01 (Fig. 2B), respectively. On the other hand, no clear zone on the glycinin-, β-conglycinin-, CMC-, corn starch-, and xylan-supplemented agar plates of *S. putrefaciens* L4 control was observed (Fig. 2A).

### L5 exhibits antimicrobial activity

**Fig 3:**
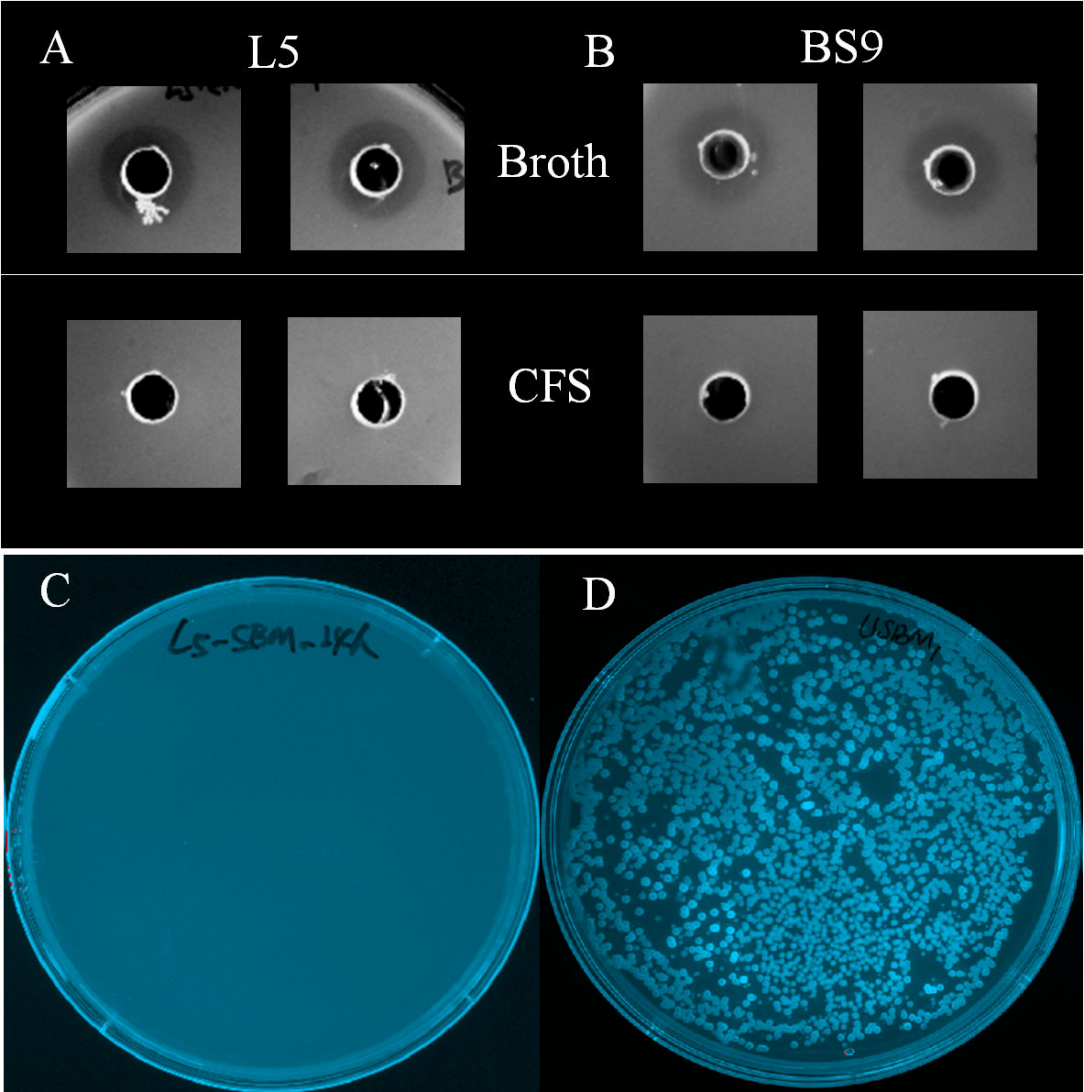
The inhibitory activity of *Bacillus subtilis* L5. (A) *E. coli* agar plates with L5 culture broth or L5 CFS. (B) *E. coli* agar plates with *B. subtilis* BS9 culture broth or BS9 CFS. (C) MacConkey plate culture result of fermented SBM (F-SBM) with L5 at 37°C for 24 hours. (D) MacConkey plate culture result of spontaneous F-SBM with sterilized MQ water at 37°C for 24 hours.

After 12 hours of aerobic incubation in the agar spot assay, the clear inhibition zone on *Escherichia coli*-growing agar plate was observed when co-culture with the L5 culture broth (containing both cell and supernatant; Fig. 3A) and BS9 culture broth (positive control; Fig. 3B). However, no inhibition zone has been observed when co-cultured with L5 cell-free supernatant (CFS) (Fig. 3A) and BS9 CFS control (Fig. 3B), suggesting contact inhibition of *E. coli* growth.

We next determined if L5 is capable of eliminating Gram-negative bacteria using the MacConkey plate, which only allows Gram-negative bacterial growth. As shown in Fig. 3C and D, there was no Gram-negative bacterial growth observed in SBM after 24 hours of fermentation with L5 (Fig. 3C), while numerous Gram-negative bacteria were observed in SBM after 24 hours spontaneously fermented with MQ water on the MacConkey plates (Fig. 3D).

**Fig 4:**
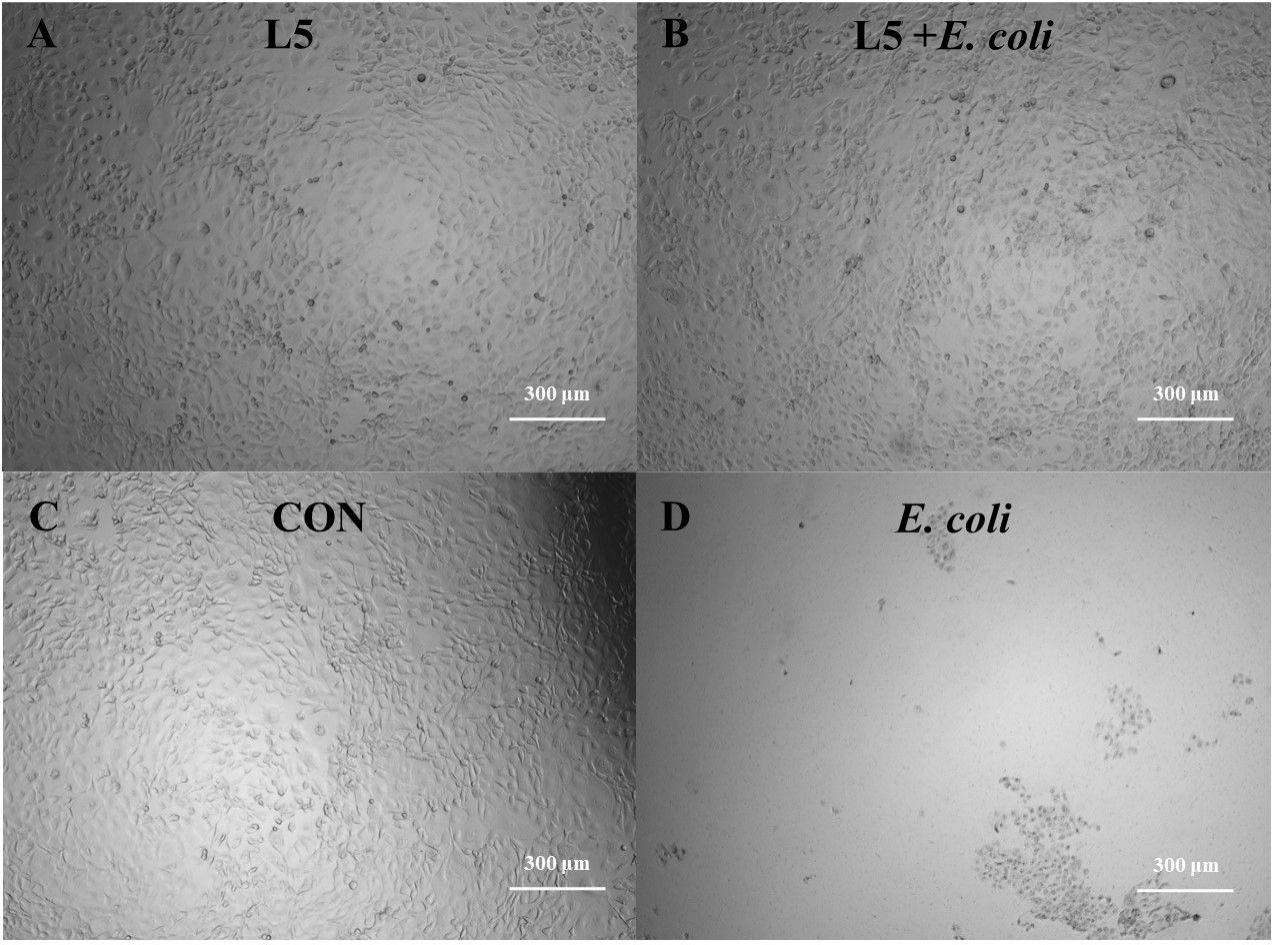
Cytotoxic effect of *Bacillus subtilis* L5 and *E. coli* on IPEC-J2 cells. (A) The IPEC-J2 cell morphology after co-culture with L5 for 6 hours. (B) The cell morphology after co-culture with L5 and *E. coli*. (C) The cell morphology in control group without bacterial co-culture. (D) The cell morphology after co-culture with *E. coli*. Morphology of the cells was captured using Cytation 5 multimode plate reader at 40× magnification in a 300-µm scale in bright-field mode.

To study if L5 can protect the intestine cell from pathogenic infection, IPEC-J2, a porcine intestine cell line was cultured in the absence and presence of L5 and *E. coli*, respectively, and in combination for 6 hours, as shown in Fig. 4. After 6 hours of aerobic incubation, no morphological changes were observed when IPEC-J2 cells were incubated with L5, L5 + *E. coli* compared with the control (CON) (Fig. 4A through C), while severe cell damage was observed when incubated with *E. coli* (Fig. 4D), suggesting L5 was capable of protecting IPEC-J2 cells from *E. coli* infection.

### Fermentation using L5 improved the SBM nutrient value

**Fig 5:**
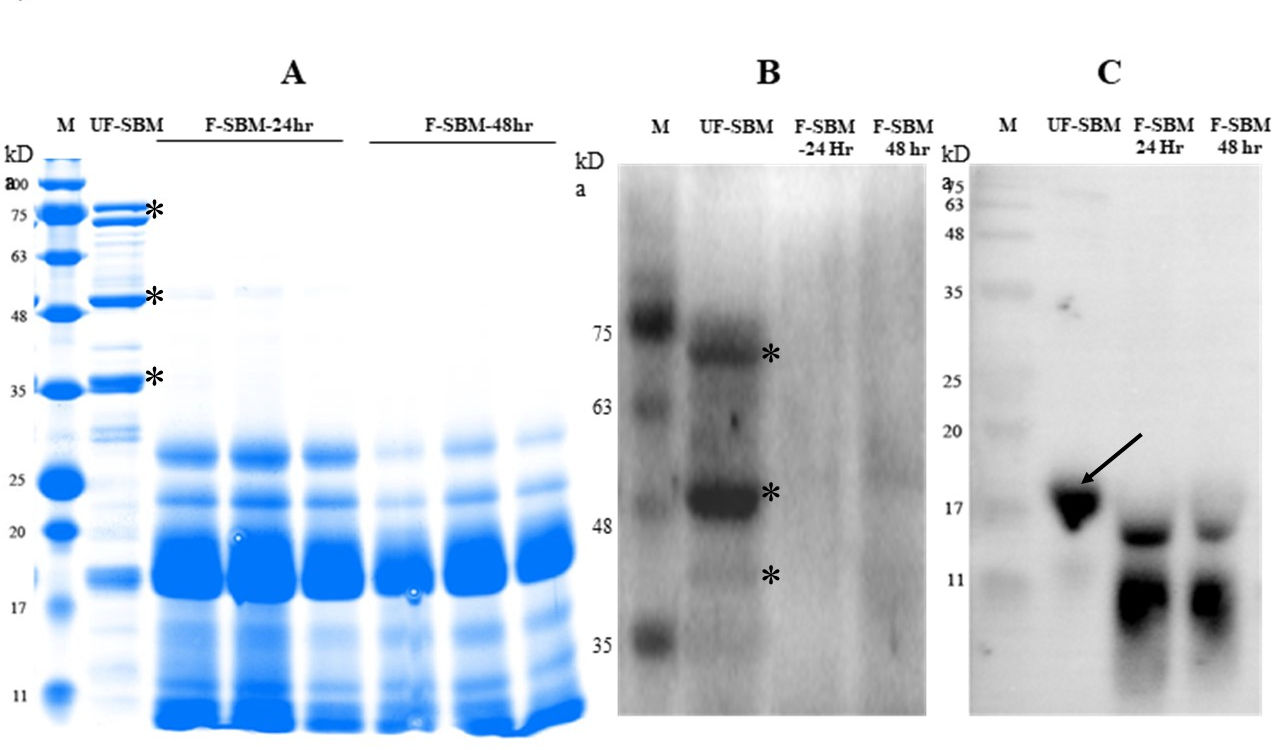
Anti-nutritional factors analysis of UF-SBM and F-SBM. (A) Protein profiling of F-SBM using SDS-PAGE in which 20 µg of protein was loaded for each well. Lane 1, protein ladder; Lane 2, UF-SBM was used as control; Lane 3–5, F-SBM for 24 hours; Lane 6–8, F-SBM for 48 hours. (B, C) Western blot detecting soy allergens in the UF-SBM and F-SBM products. Twenty micrograms of protein was loaded to each well and incubated with pig serum (1:500) (**B**) or anti-trypsin inhibitor antibody (primary antibody, 1:2,000) (**C**) followed by rabbit anti-pig IgG (1:5,000) (**B**) or anti-rabbit IgG (secondary antibody, 1:5,000) (**C**) . Lane 1, protein ladder; Lane 2, UF-SBM; Lane 3, F-SBM for 24 hours; Lane 4, F-SBM for 48 hours. Glycinin and β-conglycinin proteins are denoted as *; trypsin inhibitor is denoted as ↙.

To investigate the feasibility of L5 as a potential bacterium to improve the SBM nutrient value, SBM fermentations were performed for 24 and 48 hours, respectively. To detect the allergenic proteins including glycinin, β-conglycinin, and trypsin inhibitors of F-SBM and unfermented SBM (UF-SBM), SDS-PAGE and western blot analysis were performed and the results of SDS-PAGE showed that the subunits of glycinin (22 ~ 40 kDa) and β-conglycinin (52 ~ 76 kDa) of SBM were degraded to below 35 kDa after 24 hours or 48 hours of fermentation with L5 (Fig. 5A). Western blot results showed that the allergenic proteins (~75 kDa and ~48 kDa) could be recognized and detected in the UF-SBM samples (Fig. 5B) and the trypsin inhibitor (~17 kDa) could be recognized by anti-trypsin inhibitor antibody in the UF-SBM samples (Fig. 5C), but all allergenic proteins (~75 kDa, ~48 kDa, and ~17 kDa) were either eliminated or broken down to smaller sizes (kDa) in SBM after 24 hours and 48 hours of fermentation with L5 (Fig. 5B and C).

**Fig 6:**
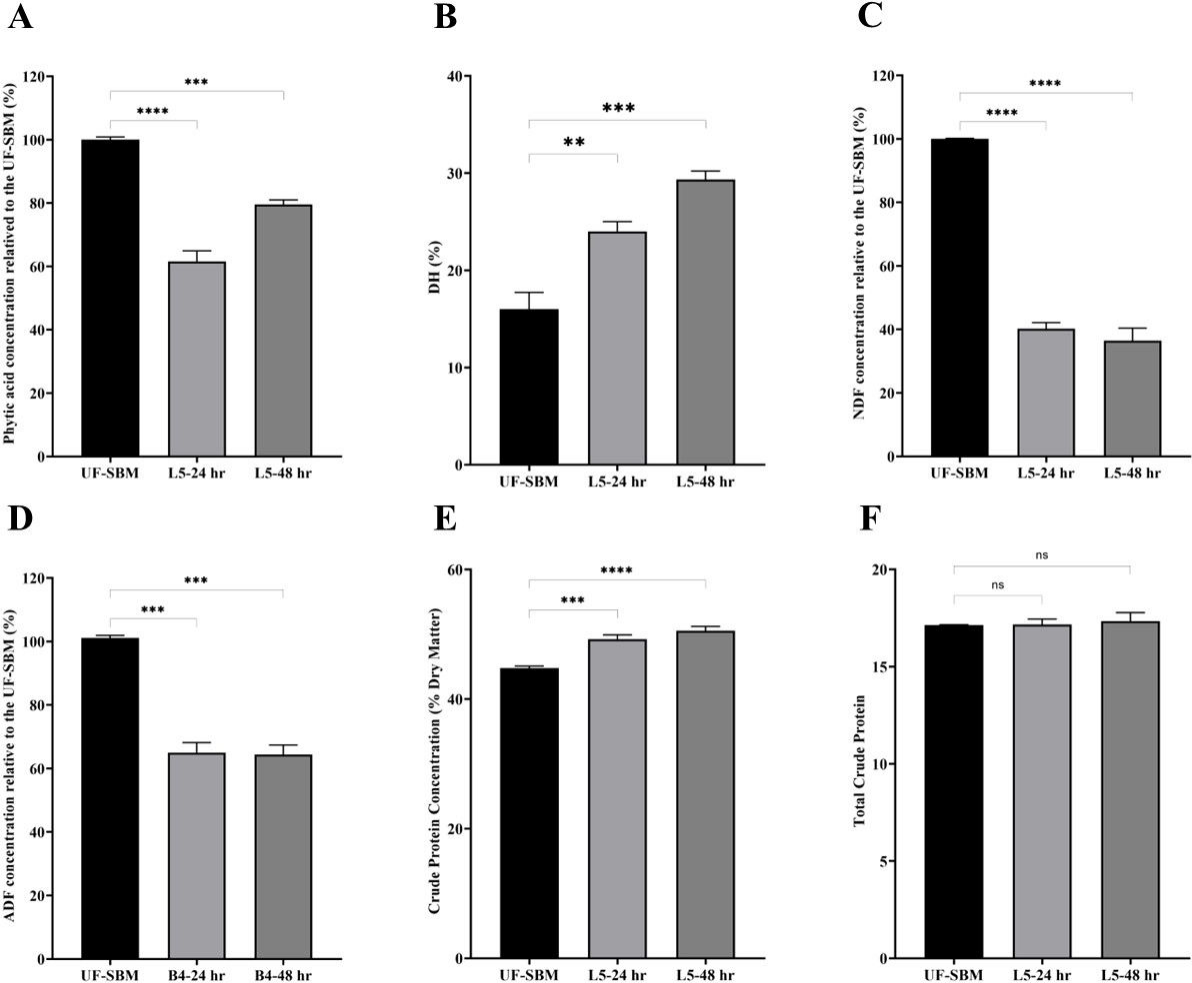
Nutritional value analysis of UF-SBM and F-SBM. (A) Phytic acid concentration of UF-SBM and F-SBM; (B) degree of protein hydrolysis (DH) analysis of UF-SBM and F-SBM; (C) neutral detergent fiber (NDF) concentration relative to the UF-SBM; (D) acid detergent fiber (ADF) concentration relative to the UF-SBM. UF-SBM was used as control; (E) crude protein concentration (% dry matter) of UF-SBM and F-SBM; (F) total crude protein (% dry matter) UF-SBM and F-SBM; B4-24 hr, F-SBM for 24 hours; B4-48 hr, F-SBM for 48 hours. Data are presented as mean ± standard error of the mean. Bars with statistical significance are denoted as **P < 0.01, ***P < 0.001, and ****P < 0.0001 using ordinary one-way analysis of variance (ANOVA). All experiments were conducted in triplicate.

We also performed experiments to determine the ability of L5 to degrade phytate, a strong antinutritional factor in SBM. The analysis results of phytic acid in UF-SBM and F-SBM showed that there are 38.42% ± 3.36% (24 hours) (*P* < 0.0001) and ~20.42% ± 1.43% (48 hours) reduction (*P* < 0.001) of phytic acid in SBM fermentation with L5 (Fig. 6A).

The analysis results of DH in UF-SBM and F-SBM showed that the DH of SBM fermented with L5 for 24 hours and 48 hours increased significantly to 24.00% ± 1.00 % (*P* < 0.01) and 29.33% ± 0.88% (*P* < 0.001), respectively, compared with 16.00% ± 1.73 % of UF-SBM (Fig. 6B).

The results of NDF, ADF, and crude protein are shown in Fig. 6C through E. The NDF of F-SBM decreased significantly by 40.23% ± 1.91% (*P* < 0.0001, 24 hours) and 36.43% ± 3.99% (*P* < 0.0001, 48 hours) (Fig. 6C), and the ADF of F-SBM decreased significantly by 65.02% ± 3.15% (*P* < 0.001, 24 hours) and 64.38% ± 3.03% (*P* < 0.001, 48 hours) (Fig. 6D), compared with the UF-SBM. The crude protein concentration of SBM fermented with L5 for 24 hours and 48 hours increased significantly to 49.25% ± 0.66% (*P* < 0.001) and 50.57% ± 0.65% (*P* < 0.0001), respectively, compared with 44.77% ± 0.31% of UF-SBM (Fig. 6E), and the total crude protein has no difference in F-SBM and UF-SBM (Fig. 6F).

## DISCUSSION

Microbial fermentation is a long-established strategy for the reduction or elimination of anti-nutritional factors as well as the improvement of nutritional value of feeds for livestock, poultry, and aquaculture. However, high temperatures (~50°C) produced by large-scale fermentation or microbial contamination during fermentation are one of the bottlenecks limiting the application of the SBM microbial fermentation (10). Therefore, screening probiotic strains with high enzymatic activity at relatively high temperatures could be advantageous for the soybean meal fermentation process. Previous studies have shown that the extracellular enzymes secreted by *B. subtilis* LYN12 exhibit significantly higher activity during submerged fermentation at 45°C compared with 37°C (11). In addition, a high yield of *B. subtilis* proteinase was found at both 40°C and 50°C (12), suggesting the potential of *B. subtilis* as an ideal probiotic for soybean meal fermentation given its broad temperature range for high enzymatic activity. Moreover, it was observed that *B. subtilis* is capable of utilizing a variety of carbohydrates such as sucrose, raffinose, and stachyose, present in soybean meal as energy sources for its propagation and metabolic activities (13, 14). The consumption of these carbohydrates resulted in the production of lactic acid and other organic acids that effectively decrease the pH during soybean meal fermentation. Lower dietary pH could alleviate the intestinal burden and stress on early-weaning piglets (15) further underscoring the significance of employing *B. subtilis* in soybean meal fermentation. In this study, we identified a novel thermophilic strain of *B. subtilis* L5, capable of thriving at varying temperatures, namely, 25°C, 37°C, and 50°C, and entering the log phase in 2 hours of culturing at 37°C and 50°C, indicating a broad temperature tolerance. The results of solid-state fermentation with L5 revealed that this probiotic could effectively digest or eliminate the anti-nutritional factors present in SBM at 37°C for 24 hours, demonstrating the rapid growth and fermentation characteristics of L5, which could ultimately shorten the fermentation duration.

Mesophilic bacteria are widely used in SBM fermentation, but most of the pathogenic microorganisms such as *E. coli*, *Salmonella* spp., and *Staphylococcus* spp. are mesophiles which can compete with the growth of the intended microorganism during fermentation (16). Although sterile SBM can prevent contamination of unwanted microorganisms, the high-energy cost of the sterilization procedure has limited its application for SBM fermentation. In this study, a potential probiotic L5 with notable antimicrobial activity was identified. The antimicrobial assay conducted on agar plates revealed that L5 exhibited a robust inhibitory effect against *E. coli* and it also inhibited the growth of Gram-negative bacteria during SBM fermentation. Additionally, on the IPEC-J2 cell model of *E. coli* infection, co-culturing with L5 revealed its ability to protect IPEC-J2 cells from *E. coli* infection, suggesting that L5 could be considered a candidate probiotic to benefit intestinal health. The observed inhibitory effect of L5 on undesirable bacterial growth during fermentation and its anti-*E*. *coli* properties could be attributed to the various antimicrobial components produced by *B. subtilis* such as bacteriocins, tetracycline, and polyenes (17). These compounds possess the ability not only to inhibit the proliferation of undesired microorganisms during fermentation but also to serve as anti-pathogen infection drugs as well as growth promoters *in vivo* in the livestock industry (18). Nevertheless, the specific mechanism through which L5 represses the growth of contaminated bacteria still remains unclear and worthy of further investigation.

Seventy to eighty percent of contents in SBM is considered anti-nutritional factors, namely, glycinin, β-conglycinin, trypsin inhibitor, and phytic acid, all of which can negatively impact intestinal health and nutrient absorption (2). It is of great importance to screen microorganisms with high enzymatic activity to effectively digest these ANFs in the SBM. Agar plates supplemented with different ANFs are widely employed for screening the proteinase, cellulase, amylase, and xylanase of bacteria (5). In this study, L5 demonstrated the capability to degrade glycinin, β-conglycinin, CMC, corn starch, and xylan, highlighting its potential as a candidate microbial strain for ANF digestion and an ideal candidate for SBM fermentation. Additionally, SDS-PAGE and WB analysis of F-SBM and UF-SBM revealed L5’s ability to digest the allergenic proteins in SBM, specifically glycinin and β-conglycinin, into smaller peptides after 24 and 48 hours of fermentation. WB results also indicated a reduction in size of the trypsin inhibitor by L5 fermentation. Moreover, phytic acid, another important anti-nutritional factor in SBM that impedes mineral absorption in the gastrointestinal tract, was reduced by approximately 20%–30% following fermentation with L5. A crucial criterion for assessing the effectiveness of fermentation is the enhancement of nutrient quality, which entails an increase in free amino acids/crude proteins and a decrease of fibers in the case of SBM. The DH refers to the percentage of peptide bonds that are degraded during protein hydrolysis and is calculated by dividing the amount of free amino acid by the total number of peptide bonds in soluble proteins (19). A previous study has reported that the DH of SBM fermented for 72 hours with *A. oryzae* AO3042 and *B. subtilis* SB102 was 21.72% and 21.25%, respectively (20), while the DH of SBM fermented for 62.32 hours with *B. subtilis natto* under optimum conditions was 15.96% (21). In our study, the DH of SBM fermented with L5 increased from 16.00% to 24.00% (24 hours) or 29.33% (48 hours), suggesting that L5 is more effective in digesting proteins into free amino acid via fermentation. In addition to this, our finding that SBM crude protein concentrations were increased from 44.8% to 49.3% and 50.6% at 24 and 48 hours of fermentation with L5, respectively, further confirms the ability of L5 fermentation to improve nutrient value. NDF and ADF are two important indicators of SBM fiber content, which could be digested by fermentation with a high cellulase-producing microbial. In the current study, L5-fermented SBM showed a significant reduction in NDF and ADF, which is in agreement with the finding of a previous study on SBM fermentation with *B. subtilis* and *A. oryzae* (22) and the study on rapeseed meal and canola meal fermentation (23). Thus, L5 is a promising candidate fermentation probiotic for improving the nutrient value of feedstuff.

In summary, a novel antimicrobial thermophilic strain of *B. subtills* L5 with antimicrobial properties was isolated and characterized in the present study. Fermentation with L5 effectively degraded various anti-nutritional factors in SBM, including glycinin, β-conglycinin, trypsin inhibitor, phytic acid, NDF and ADF, which ultimately led to a significant improvement in the crude protein and free amino acid concentration. Our findings on the probiotic and fermentation characteristics of L5 suggest that this novel microbial with dual function could be a promising candidate microorganism for feed fermentation to improve its nutrient values.

## MATERIALS AND METHODS

### Bacterial culture conditions

*B. subtilis*, “BS9” which was isolated previously and stored in our lab at University of Guelph, Canada, was used as a positive control. K88 (F4)-expressing Enterotoxigenic *Escherichia coli* used in this study was acquired from Animal Health Lab, University of Guelph, Canada. All cultures were performed using Luria-Bertani (LB) liquid broth or on LB agar plates in 37°C shaking incubator under aerobic conditions.

### Sample collection and bacteria identification

The lake mud sample was taken from the shore of the Little Manitou Lake, which is known to have high salinity content, in Saskatchewan, Canada. To perform bacterial screening on the lake mud sample collected, 5 g of the sample was first suspended in 50 mL of sterile PBS followed by vortexing for 10 seconds. Particulate material was then removed by centrifuging at 400 × *g* for 2 min. The supernatant was then diluted 10 times with PBS. Finally, 100 µL of diluted suspension was plated on LB agar plates and incubated at 37°C for 24 hours. Twenty-four bacterial colonies with different morphologies were purified and submitted to the Animal Health Laboratory at the University of Guelph for species identification using a matrix-assisted laser desorption ionization-time of flight mass spectrometry (MALDI-TOF MS, Bruker, Canada). Five isolates of potential probiotics, including *Bacillus* spp. L1, *Bacillus cereus* L2, *Bacillus pumilus* L3, *Shewanella putrefaciens* L4, and *B. subtilis* L5, were selected for temperature tolerance analysis.

### Growth characteristics analysis

To screen for thermophilic strains of bacteria, the growth of *Bacillus* spp. L1, *B. cereus* L2, *B. pumilus* L3, *S. putrefaciens* L4, and *B. subtilis* L5 were tested using optical density measurements at 600 nm (OD600), and the growth curve was plotted based on the OD600 value. Briefly, 60 µL of each overnight culture of the above bacteria was incubated with 6 mL of LB broths at 50°C for 12 hours, and OD600 of bacteria was read every 2 hours over a period of 2 ~ 12 hours at 37°C by using a Cytation 5 multimode plate reader (Biotek, Winooski, VT).

To analyze the temperature tolerance, the thermophilic strain *B. subtilis* L5 was incubated at 25°C, 37°C, 50°C, and 60°C for 12 hours, and the growth curve of L5 was obtained using OD600 measurements. Each experiment was performed in triplicates.

### Enzymatic activity analysis

LB agar plates containing glycinin, β-conglycinin, carboxymethyl cellulose (CMC) (Acros Organics, USA), corn starch, and xylan (Sigma Aldrich, USA) were used to test the protease, cellulase, amylase, and xylanase activities of *B. subtilis* L5. The glycinin and β-conglycinin were isolated from soybean meal as described by Li et al. (5), and the glycinin or β-conglycinin agar plates were prepared by adding 50 mL of glycinin or β-conglycinin and 1.5 g of agar to 50 mL of LB. CMC agar plates were prepared by dissolving 1 g of CMC and 1.5 g of agar in 100 mL of LB. Corn starch agar plates were prepared by dissolving 1 g of corn starch and 1.5 g of agar in 100 mL of LB. Xylan agar plates were prepared by dissolving 0.3 g of xylan and 1.5 g of agar to 100 mL of LB. All broth was autoclaved for 25 min at 121°C before dispensing to the petri dishes.

A single colony of *B. subtilis* L5 was cultured in LB broth overnight at 37°C, and then, 1 µL of L5 culture broth was inoculated on the agar plates containing glycinin, β-conglycinin, CMC, corn starch, or xylan. The CD and HD were recorded after 12 hours of culturing at 37°C; then, HC was calculated as the ratio of the HD and CD. Each experiment was performed in triplicate.

### Antimicrobial activity detection

The inhibitory activity of L5 was evaluated using an agar spot assay as previously described by Sudan et al. with minor modifications (24). Briefly, once the autoclaved LB agar cooled down to approximate 50°C, 1 × 10^8^ CFUs of *E. coli* were added into 20 mL of autoclaved LB agar, and then, this mixture was dispensed into a petri dish. After the agar mixture had solidified, holes of approximately 8 mm in diameter were punched in. Subsequently, 10 µL of various solutions, the L5 culture broth, CFS of L5, CFS of BS9 (serving as a positive control), or BS9 culture broth were added to the holes. The plates were then incubated at 37°C under aerobic conditions. After 12 hours of incubation, the diameters of the inhibition zones were measured and analyzed.

Investigations were conducted to assess whether L5 confers a protective effect on intestinal cells subjected to *E. coli* damage using IPEC-J2 cells, an intestinal porcine enterocyte cell line derived from the jejunum of a piglet. The protective efficacy of L5 against *E. coli* on IPEC-J2 cells was evaluated as previously described with minor modification (25). Briefly, IPEC-J2 cells were seeded at a density of 4 × 10^5^ cells/mL (100 µL for each well) in a 96-well plate (Corning, Fisher Scientific, Mississauga, Canada) and incubated for 24 hours, to allow full confluence to the bottom of the plate. The cells were washed with pre-warmed PBS twice and placed in fresh CO_2_-independent media supplemented with 10% FBS, followed by a minimum incubation of 30 min. Subsequently, IPEC-J2 cells were incubated with 1 × 10^8^ CFU/mL (100 µL for each well) of L5 or LB (100 µL for each well), with or without 1 × 10^7^ CFU/mL (100 µL for each well) of *E. coli*, and incubated at 37°C for 6 hours. The morphology of the cells was visualized and captured using a Cytation 5 multimode plate reader (Biotek, Winooski, VT, USA) at a 40× magnification and a 300-µm scale in bright-field mode.

### Soybean meal solid fermentation

L5 was prepared by incubating a single colony in LB broth at 37°C (200 RPM) overnight and diluted to ~2.0 × 10^7^ CFU/mL. Thirty grams of SBM were mixed with 30 mL of L5 culture broth (~2.0 × 10^7^ CFU/mL) in an aluminum box. After incubating at 37°C for 24 or 48 hours, the F-SBM samples or UF-SBM were dried at 60°C for 24 hours.

To detect the growth of contaminant bacteria, 1 g of F-SBM samples was collected at 24 hours prior to drying and mixed with 10 mL of sterilized Milli-Q (MQ) water. After vortexing for 30 seconds, 100 µL of resuspended solution was spread on MacConkey agar (Fisher Scientific, Mississauga, Canada) plates and cultured at 37°C overnight, while spontaneously fermented SBM with MQ water for 24 hours was used as a control.

### Chemical analysis of fermentation soybean meal

Glycinin, β-conglycinin, and trypsin inhibitors were detected as previously described, with minor modification (26). Briefly, 0.5 g of dried F-SBM (24 hours or 48 hours) or UF-SBM was suspended in 5 mL of PBS and ultra-sonicated for 30 seconds on ice. Then, the supernatant was collected after centrifuging at 7,000 × *g* for 10 min at 4°C. Twenty micrograms of the supernatant was loaded onto a 14% SDS-PAGE for soluble protein detection through electroporation. Images were taken using ChemiDoc XRS+ (Bio-Rad, USA) and analyzed with Image Lab software. After gel electrophoresis, the samples were transferred to a polyvinyl difluoride membrane (Millipore, Billerica, USA) using the Trans-BlotR Turbo transfer system (Bio-Rad, USA). Membranes were blocked in TBS-T with 5% skim milk powder at room temperature for 1 hour and then incubated with pig serum (1:500 dilution) or Rabbit Anti-Trypsin inhibitor antibody (1:2,000 dilution, ab34549, Abcam, Cambridge, UK) as the primary antibody at 4°C overnight. Pig serum was collected from 8-week-old piglets that were previously exposed to feed containing SBM for 28 days and had developed an immune response to SBM allergens. Rabbit Anti-Pig IgG (1:5,000, ab6777, Abcam, Cambridge, UK) or Anti-Rabbit IgG (1:5,000 dilution, HRP linked; Cell Signaling Technology, USA) were used as secondary antibodies. The protein density was detected by the Clarity Western ECL substrate and imaged with the ChemiDoc XRS+ System (Bio-Rad, USA).

The phytic acid content of F-SBM (24 hours and 48 hours) was analyzed by the colorimetric method as previously described, with minor modification (27). Briefly, 1 g of UF-SBM or F-SBM was added to 20 mL HCl 2.4% (m/V) and mixed for 16 hours at room temperature, followed by centrifuging at 4,000 × *g* for 10 min at 10°C. The supernatant was mixed with 2 g of NaCl at 350 RPM for 20 min at room temperature and was allowed to settle at 4°C for 60 min, followed by centrifuging at 4,000 × *g* for 20 min at 10°C. Ten microliters of supernatants from different samples were mixed with MQ water and added to a well of a 96-well plate. An equal volume of MQ water was tested as a control, while phytic acid (TCI America) was used as standard. Thirty microliters of Wades reagent (0.03%, m/V of iron (III) chloride hexahydrate with 0.3%, m/V of sulfosalicylic acid, Thermo Scientific, USA) was added to wells, and absorbance was measured immediately at 500 nm using a Cytation 5 multimode plate reader (Biotek, Winooski, VT, USA).

The degree of protein hydrolysis was analyzed using the o-phthaldialdehyde (OPA) assay, as previously described by Nielsen et al. (28), with minor modifications. Briefly, the total amino acid of 0.5 g sample was dissolved using 10 mL of 6 M of HCl at 110°C for 24 hours, and then, the free amino acid of the 0.5-g sample was collected after dissolving in the MQ water. The supernatant of samples was then collected after centrifuging at 14,000 × *g* for 20 min at room temperature. A total of 20 µL supernatant of each sample was mixed with 300 µL of the OPA reagent for further absorbance measurement after 2 min of incubation. The absorbance was measured at 340 nm using a Cytation 5 multimode plate reader (Biotek, Winooski, VT, USA) and using MQ water as the control. The DH was calculated using the formula below. DH (%) = $\frac{NH2(free)}{NH2(total)}$ × 100%, where NH_2_ (free) is the concentration of free amino acid of the samples, and NH_2_ (total) is the total amount of amino acid of the samples after being hydrolyzed in 6 M HCl.

The dry matter was determined according to standard operating procedures method 930.15 (Association of Official Analytical Chemists, AOAC, 2005). The crude protein was determined using the DUMAS method (AOAC, 1997) following the manufacturer’s protocol of the LECO Protein/Nitrogen Analyzer (LECO Corp., USA); the NDF and ADF were analyzed with an Ankom2000 Fiber Analyzer (Ankom Technology) following ANKOM technology procedure 13.

### Statistical analysis

All experiments were conducted in triplicate, and the data were analyzed using one-way ANOVA with treatment using a GraphPad Prism software version 9.0. Data were considered significant at *P* < 0.05.

## Data Availability

All relevant data were included in the paper.
